# Corporate whistleblower system and workplace safety performance: from the dual perspectives of safety investment and internal control

**DOI:** 10.3389/fpubh.2026.1935312

**Published:** 2026-09-23

**Authors:** Lin Liu, Xingcheng Zhao, Jinnan Wu

**Affiliations:** 1 School of Management Science and Engineering, Anhui University of Technology, Ma’anshan, China; 2 School of Business, Anhui University of Technology, Ma’anshan, China

**Keywords:** accident prevention, internal control quality, safety investment, safety performance, whistleblower system, whistleblowing

## Abstract

**Introduction:**

While corporate whistleblower system helps prevent and mitigate corporate violations within business operations, yet its effectiveness in the field of workplace safety remains largely unsubstantiated. Thus, it is both theoretically and practically important to examine whether and how the adoption of whistleblower system is associated with workplace safety performance.

**Methods:**

Grounded in an integrated framework of accident causation theory (explaining the direct effect on safety performance), deterrence theory (elucidating the mediating role of safety investment), and signaling theory (elucidating the mediating role of internal control quality), this study examines the effect and underlying mechanisms of corporate whistleblower system adoption on workplace safety performance. Using a sample of 3,086 firm-year observations from 266 A-share listed companies in high-risk industries, we employ fixed-effects models and mediation analyses to investigate the relationship between corporate whistleblower systems and workplace safety performance and the underlying mechanisms.

**Results:**

The results show that whistleblower system adoption is associated with significant improvements in safety performance, as evidenced by reductions in workplace fatalities and employee mortality rates. This finding remains robust after a series of endogeneity corrections and robustness checks. Mechanism tests further reveal two distinct pathways: a resource pathway via increased safety investment and a governance pathway via enhanced internal control quality. Heterogeneity analyses indicate that the positive correlation between corporate whistleblower system adoption and safety performance is particularly pronounced in firms located in eastern China.

**Discussion/Conclusion:**

This research not only extends the governance scope of whistleblower systems from financial and compliance domains to workplace safety, but also highlights the critical role of internal employees as safety governance actors. The findings offer policy implications for designing a collaborative internal-external safety governance system that leverages both regulatory oversight and internal self-regulation.

## 1 Introduction

Workplace safety is fundamental to both human well-being and economic prosperity (1). However, contemporary workplaces are becoming increasingly dynamic and uncertain (2), a trend which has intensified the complexity of occupational health and safety risks (3). Failure to identify and contain safety hazards in a timely manner can result in major accidents with severe social and economic costs (4). In China alone, nearly 20,000 workplace accidents occurred in 2025, resulting in over 18,000 fatalities. These figures remind that existing hazard identification and mitigation mechanisms remain insufficient. This calls for a deeper understanding of how firms can effectively surface and act upon safety-related information from within their organizations.

The safety risks inherent in the production processes of high-risk industries are characterized by their specialized, concealed, and dynamic nature, making them difficult to detect through external oversight alone (5). Internal employees, however, operate at the front line of production and possess unique informational and cognitive advantages, enabling them to identify and report hazards at the earliest possible stage (6). Internal whistleblowing, a governance arrangement that motivates voluntary employee disclosure, has proven effective in detecting corporate fraud (7), reducing major operational risks (8), and mitigating negative economic consequences (9). Thus, Chinese regulators have actively incentivized enterprises to establish whistleblower systems. Yet, despite these policy efforts, empirical evidence on whether and how such systems are associated with workplace safety performance remains scarce.

A growing body of literature has examined the consequences of whistleblowing, yet findings remain mixed and context-dependent. On the one hand, whistleblowers play a critical role in expediting the exposure of misconduct, preserving organizational integrity, and safeguarding the public interest (10). Corporate whistleblower system serves as a negative feedback mechanism that exposes internal corruption (11), reduces financial fraud risks (12), and helps firms contain losses and avoid severe economic damage (9). On the other hand, whistleblowing may also trigger adverse consequences by disrupting organizational equilibrium—eroding employees’ sense of group identity, undermining morale, and impairing organizational performance (13). Moreover, the effectiveness of whistleblower system varies considerably across institutional contexts: food safety whistleblowing schemes have fallen short of their intended oversight goals (14), while similar mechanisms in capital markets have been found to generate adverse organizational cultures (15).

Despite suggestive evidence from other domains, three critical gaps remain in the existing literature. First, extant studies have largely concentrated on the consequences of whistleblowing in financial misconduct and corporate governance domains (7, 11, 12), leaving workplace safety, a domain equally reliant on internal information disclosure and reporting, largely unexplored. Safety hazards in high-risk industries are characterized by their specialized, concealed, and dynamic nature, making them resistant to external oversight alone (5). Yet whether the governance logic of whistleblowing can transfer to the safety domain, and through what mechanisms, remains an open question. Second, prior safety research has predominantly emphasized external regulators (government and safety agencies) as safety drivers (16), overlooking the governance potential of internal employees (6, 17). Frontline workers operate at the production frontier and possess inherent informational advantages in detecting hazards early, yet this human capital has not been sufficiently mobilized for safety governance. Third, the mechanisms through which whistleblowing is associated with safety performance have not been theorized or tested. While safety rewards (18) and formal or informal punishments (19) have received attention as incentives for employee safety participation, whether and how a structured whistleblower system, as an institutionalized information channel, can improve safety outcomes through specific pathways remains theoretically and empirically unresolved. This gap constrains our understanding of the full pathway from deterrence to accident prevention.

To address these gaps, this study integrates accident causation theory (20, 21), deterrence theory (22, 23), and signaling theory (24, 25) into a unified analytical framework, and examines the relationship between whistleblower systems and workplace safety performance using a sample of A-share listed companies in Chinese high-risk industries from 2013 to 2024. Specifically, we seek to answer: Is whistleblower systems associated with workplace safety performance? If so, how do the resource pathway and governance pathway operate? In doing so, this study makes three contributions. First, it extends whistleblowing research from financial and compliance domains to workplace safety, revealing how the distinct characteristics of safety hazards—being concealed, dynamic, and technically specialized—reshape the operational logic of whistleblower governance. Second, it opens the “black box” between whistleblowing and safety outcomes by identifying and testing two distinct pathways—a resource pathway (safety investment) and a governance pathway (internal control quality). Third, it integrates three theoretical perspectives into a coherent pathway, offering a more complete explanatory framework than prior single-theory approaches.

## 2 Theoretical framework and research hypotheses

### 2.1 A unified analytical framework for understanding workplace whistleblowing

This study constructs an integrated theoretical framework that incorporates accident causation theory (20, 21), deterrence theory (22, 23), and signaling theory (24, 25) into a unified pathway. The three theories explain different stages of the causal process: signaling theory explains why employees report hazards; deterrence theory explains why firms respond; and accident causation theory explains why responses prevent accidents.

Front-end: Signaling and employee whistleblowing behavior. Signaling theory suggests that signals can reduce information asymmetry by conveying credible information from informed to uninformed parties (24, 25). In the context of workplace safety governance, management possesses decision-making authority over safety resource allocation but lacks direct perception of on-site hazards; employees possess first-hand hazard information but often remain silent for fear of retaliation. This information asymmetry lies at the root of safety governance failures. Whistleblower systems convey a credible signal of organizational safety commitment to employees by establishing formal, confidential whistleblowing channels and anti-retaliation protections. This signal reduces employees’ fear of whistleblowing consequences and encourages them to proactively identify and report hazards (26). In this sense, whistleblower systems transform employees from passive observers into proactive safety sentinels (11).

Mid-stream: Deterrence effects and organizational response. Deterrence theory posits that increasing the certainty and severity of sanctions can deter violations (22, 23). When employees report hazards through whistleblower channels, management gains critical information about safety vulnerabilities. At this point, potential regulatory sanctions, reputational losses, and legal liabilities constitute the expected costs of inaction. Driven by these deterrence effects, management is incentivized to take corrective actions. It should be noted that the deterrence effect of whistleblower systems does not operate directly through external regulators, but indirectly by converting internal information into managerial decision-making pressure.

Back-end: Dual pathways of resource and governance in accident prevention. Accident causation theory holds that the root causes of accidents can be traced to unsafe acts of people, unsafe conditions of objects, and management deficiencies (20, 21). If hazards at these three levels are not promptly identified and eliminated, they ultimately lead to accidents. The corrective actions taken by firms interrupt the accident causation chain through two pathways: the resource pathway—increasing safety investment, upgrading equipment, and improving protective facilities, directly improving the safety condition of objects; and the governance pathway—strengthening internal controls, improving risk identification and compliance processes, fundamentally reducing management deficiencies. These two pathways operate in parallel, blocking the accident chain from both the resource and governance dimensions.

Based on this integrated analytical framework (see Figure 1), this study proposes the following Hypotheses.

**Figure 1:**
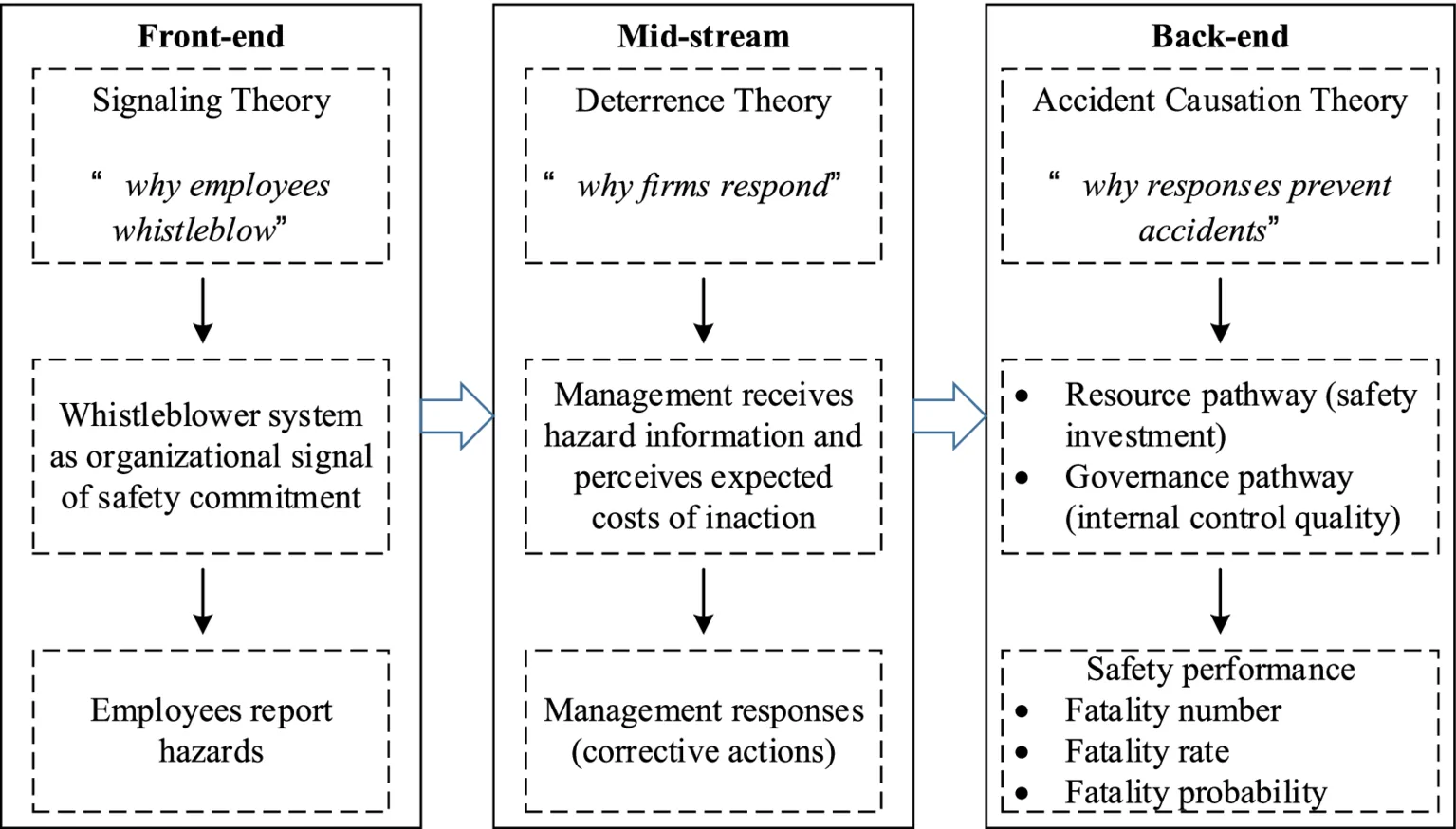
A unified analytical framework for understanding workplace whistleblowing.

### 2.2 Corporate whistleblower system and safety performance

Accident causation theory suggests that if safety hazards are not promptly identified and addressed, they ultimately lead to accidents (21). Employees in high-risk industries work on the front lines of production and possess inherent advantages in detecting and whistleblowing on safety hazards at the earliest opportunity (6, 17). By providing a safe and anonymous whistleblowing channel, corporate whistleblower system incentivizes employees to proactively identify and report potential safety hazards. Based on the hazards reported by employees, firms can precisely carry out hazard identification and remediation efforts, thereby reducing unsafe behaviors, unsafe conditions, and management deficiencies. This intervention cuts off both the direct and root causes in the accident causation chain, effectively preventing the escalation of safety risks from quantitative accumulation to qualitative change, and consequently reducing the likelihood of safety accidents. Thus, corporate whistleblower system serves as an institutional mechanism that interrupts the accident causation chain at an early stage, enhancing the timeliness and comprehensiveness of hazard detection, and ultimately reducing the probability of safety accidents. Based on the above analysis, the following hypotheses are proposed:

*H1*: Corporate whistleblower system positively correlates with safety performance.

### 2.3 The mediating roles of safety investment and internal control quality

This study selects safety investment and internal control quality as mediating variables based on the following considerations. Theoretically, safety investment and internal control quality are the most direct organizational responses triggered by whistleblower information. The former reflects resource reallocation (addressing “unsafe conditions of objects”), while the latter reflects process optimization (addressing “management deficiencies”). Thus, safety investment and internal control quality correspond, respectively, to the “object-related” and “human/management-related” accident chains in accident causation theory (20, 21), achieving higher theoretical alignment. Moreover, these two pathways directly correspond to two core policy tools in China’s work safety regulation—the safety fund appropriation system and internal control standards, which offer clear practical implications and yield actionable evidence for policy optimization.

The above theoretical framework posits that whistleblower systems convey credible signals of organizational safety commitment to employees, thereby encouraging hazard whistleblowing. This study therefore focuses on the organizational response consequences of signaling, i.e., whether whistleblower systems prompt firms to take observable corrective actions (increased safety investment and improved internal control quality). If whistleblower systems cannot trigger observable organizational actions, their safety governance effects would not materialize; thus, testing organizational responses is a necessary condition for validating the pathway. Further, the employee-level mechanisms are inherently micro-level phenomena. While these mechanisms are theoretically important, their empirical investigation falls beyond the scope of our archival research design.

Importantly, we conceptualize safety investment and internal control quality as two parallel rather than sequential mechanisms. They operate simultaneously and independently following the activation of whistleblower systems, rather than one preceding the other in a temporal chain. Safety investment addresses the physical conditions of production through equipment upgrades, protective facilities, and safety training, thereby directly reducing the unsafe conditions that constitute one root cause of accidents. Internal control quality, in parallel, addresses the organizational and managerial dimensions through enhanced risk assessment, information communication, and compliance monitoring, thereby reducing the management deficiencies that constitute another root cause. Each pathway provides an independent channel through which whistleblower information translates into improved safety outcomes. This parallel conceptualization is consistent with the theoretical logic that whistleblower systems generate multiple simultaneous governance effects (8, 10).

Safety investment, defined as the total human, financial, and material resources a company allocates to safety activities, is a critical determinant of workplace safety performance (27). According to accident causation theory (21), increased safety investment reduces unsafe behaviors and conditions, thereby breaking the accident causation chain (28) and improving workplace safety outcomes (29). However, safety investments entail uncertain and lagged returns (30, 31), and may even depress short-term profitability (32). Consequently, when the expected costs of non-compliance are low, firms thus have incentives to underinvest in safety. Deterrence theory posits that increasing the certainty or severity of sanctions raises the cost of non-compliance and deters misconduct (22, 23). Corporate whistleblower systems increase the probability of non-compliant behavior and safety hazards being detected (the certainty of punishment) by enabling employees to report hazards, thereby raising the expected cost of non-compliance (19). From a deterrence perspective, to avoid potential financial and reputational penalties firms respond by increasing safety investments (such as upgrading equipment and installing protective facilities), which directly improve safety conditions. Through this deterrence-enhancing mechanism, corporate whistleblower systems can deter corporate violations (33). Based on the preceding analysis, the following hypotheses are proposed:

*H2*: Corporate whistleblower system positively correlates with safety performance through the mediation of safety investments.

Internal control systems enable effective organizational governance by ensuring that information flows across hierarchical levels (34). Within this framework, the element of information and communication constitutes a foundational pillar (35), facilitating risk assessment and control activities (36). However, in the context of workplace safety, information asymmetry often persists: frontline employees possess firsthand knowledge of hazards yet often remain silent for fear of retaliation. This information gap impairs the risk assessment function of internal controls. Signaling theory suggests that signals can reduce information asymmetry by conveying credible information from informed to uninformed parties (24, 25). In our context, whistleblower system serves as a credible signal to employees that the organization is committed to workplace safety by establishing formal, confidential whistleblowing channels and anti-retaliation protections. This signal encourages employees to voluntarily report hazards (26), facilitating an upward flow of information (37). Consequently, this allows management to conduct targeted risk assessments and implement timely remedial actions. Thus, drawing on signaling theory, whistleblower system strengthens the information and communication component of internal controls, ultimately enhancing workplace safety performance. The subsequent hypothesis is proposed based on the preceding analysis:

*H3*: Corporate whistleblower system positively correlates with safety performance through the mediation of internal control quality.

## 3 Methods

### 3.1 Data source

Following Fisman and Wang (38), this study uses Shanghai and Shenzhen A-share listed companies from 2013 to 2024 as the research sample. Excluding the transportation sector, the final sample consists of eight high-risk industries frequently associated with safety accidents, namely: coal mining (B01), ferrous metal ore mining (B05), non-ferrous metal ore mining (B07), petroleum processing and coking (C41), manufacture of raw chemical materials and chemical products (C43), smelting and pressing of ferrous metals (C65), smelting and pressing of non-ferrous metals (C67), and civil engineering construction (E01). Given that special treatment (ST) companies are more inclined to engage in earnings management (e.g., manipulating non-recurring gains and losses) to avoid delisting, which may distort the authenticity of financial data, while delisted companies often suffer from severe financial or governance problems (39, 40), we followed existing studies (41, 42), and excluded ST and delisted companies during the observation period, which can reduce sample heterogeneity and noise interference (40). The initial total sample comprises 3,192 firm-year observations across 266 listed firms. Due to missing data in the three variables (i.e., *number of employees*, *corporate whistleblower systems*, and *firm size*), the available sample sizes were reduced to 3,182, 3,096, and 3,086, respectively. Consequently, our final regression sample comprises 3,086 firm-year observations. To ensure transparency, Figure 2 presents the detailed sample selection flow, illustrating each exclusion step and the corresponding observation counts.

**Figure 2:**
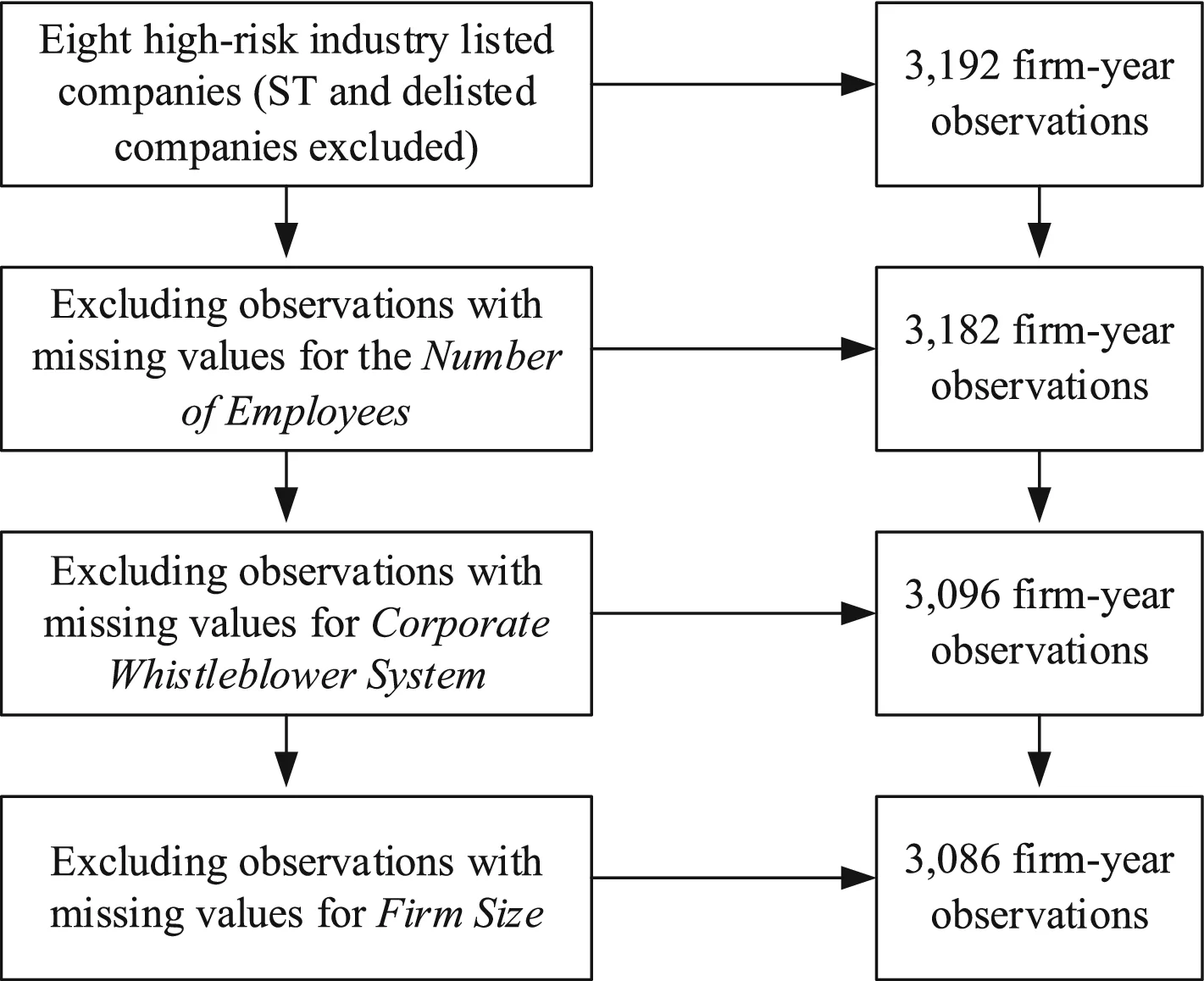
Sample screening flowchart.

Data are collected from multiple sources, including corporate annual reports, work safety accident announcements, official websites of the Ministry of Housing and Urban–Rural Development and the Ministry of Emergency Management of the People’s Republic of China, the WinGo Financial Text Data Platform, the DIBO Internal Control Database, the CSMAR Database, and the provincial statistical bulletins on national economic and social development.

### 3.2 Variable definition

#### 3.2.1 Dependent variables

Following Fisman and Wang (38), safety performance was measured with three outcome indicators: the number of workplace fatalities (*Death_N*), employee fatality rate (*Death_R*), and a dummy variable indicating the occurrence of workplace fatalities (*Death_D*). Data on the number of workplace fatalities at listed companies were collected according to the following procedure. First, we compiled workplace fatality figures disclosed in the Corporate Social Responsibility (CSR) reports, annual reports, and semi-annual reports of listed companies. Second, to verify and refine our data, we retrieved and organized casualty data from work safety accident announcements on the *Juchao Information Network*. Third, for companies in civil engineering construction (E01), we cross-referenced accident records and fatality statistics from the *Work Safety Management Committee of the Ministry of Housing and Urban–Rural Development*. Finally, any remaining data gaps were filled using Baidu searches and media reports. In this process, accidents involving a listed company or its holding subsidiaries are attributed to the parent company. Furthermore, given the high prevalence of outsourcing in the civil engineering construction industry, fatalities among non-regular employees in this sector are included in the total fatality count.

#### 3.2.2 Independent variables

Corporate whistleblower systems (*Report*). Following the measurement approach proposed by Rani et al. (43), we construct this indicator by analyzing the frequency of keywords in corporate reports. The keyword dictionary was developed through a systematic procedure combining seed word extraction, word vector expansion, iterative refinement, and manual screening with contextual co-occurrence rules to minimize false positives (see Appendix for detailed construction steps). Keyword counts were normalized by report length to account for disclosure intensity. Following standard practice, *Report* is calculated as the natural logarithm of one plus the sum of the normalized keyword frequencies.

#### 3.2.3 Mediation variables

Special Reserve balance (*Srb*). According to the *Measures for the Collection and Use of Enterprise Work Safety Funds* (Cai Zi [2022] No. 136), high-risk industry enterprises are required to appropriate work safety funds at prescribed rates and record the appropriation in the “Special Reserve” account under owners’ equity. When enterprises actually incur safety expenditures, whether expense-type (e.g., training, protective equipment) or capital-type (e.g., safety equipment purchases), the Special Reserve balance is directly reduced. Therefore, following Ma et al. (44), we use Special Reserve balance (*Srb*) as a reverse proxy for actual safety investment: a higher *Srb* indicates a lower actual safety investment. To mitigate heteroscedasticity, we use the natural logarithm of this *Srb*.

Internal control quality (*Incont*). Following Cheng (45) and Bai et al. (41), we adopt the internal control quality index in the DIBO Internal Control Database as our primary measure of internal control quality. This index provides a comprehensive and objective assessment of a firm’s internal control system, incorporating diverse corporate information to yield a scientifically grounded and systematic measure for evaluating internal control effectiveness in Chinese listed firms (45). A higher index value indicates more effective internal control outcomes (41).

#### 3.2.4 Control variables

We control for a set of firm-specific and industry characteristics, including firm size (*Size*), return on assets (*Roa*), Tobin’s Q (*Tobin Q*), debt-to-equity ratio (*Lev*), regional workplace fatalities (*Acci*), number of employees (*Empl*), revenue growth rate (*Growth*), board size (*Boardsize*), proportion of independent directors (*Indp*), and industry concentration (*HHI*). Detailed variable definitions are presented in Table 1.

**Table 1:** Variable measurement.

Variable type	Variable name	Symbol	Measurement indicators
Dependent variables	The number of workplace fatalities	*Death_N*	Natural logarithm of the number of workplace fatalities plus one
	Employee fatality rate	*Death_R*	The ratio of work-related fatalities to total employees, multiplied by 1,000
	Workplace fatalities occurrence	*Death_D*	A dummy variable equal to 1 if the firm experienced at least one work-related fatality during the year, and 0 otherwise.
Explanatory variables	Corporate whistleblower system	*Report*	Natural logarithm of the frequency of keywords related to corporate whistleblower system
Mediating variables	Special Reserve balance	*Srb*	Natural logarithm of the special reserve balance
	Internal control quality	*Incont*	Natural logarithm of Dibo Internal Control Index plus one
Control variables	Enterprise size	*Size*	Natural logarithm of total assets
	Return on Assets	*Roa*	Net income divided by total assets
	Tobin’s Q	*Tobin Q*	The ratio of the market value of the company to the replacement cost of its assets
	Debt-to-Equity Ratio	*Lev*	The ratio of total liabilities to total assets
	Number of employees	*Empl*	Number of employees at year-end
	Regional workplace fatalities	*Acci*	The natural logarithm of the number of fatalities from work-related accidents in the province where the firm is located.
	Revenue growth rate	*Growth*	The growth rate of operating revenue compared to the previous year.
	Board size	*Boardsize*	Natural logarithm of the total number of board members plus one
	Proportion of Independent Directors	*Indp*	The number of independent directors / Total number of board members
	Industry Competition Level	*HHI*	The sum of the squares of the ratios of each firm’s revenue to total industry revenue

### 3.3 Model specification

To examine the relationship between corporate whistleblower system and safety performance, model 1 presents the baseline regression as follows:

$${\text{Prosafety}}_{\mathrm{it}}=\alpha_{0}+\beta_{1}{\text{Report}}_{\mathrm{it}}+\beta_{2}{\text{control}}_{\mathrm{it}}+{\text{Firm}}_{i}+{\text{Year}}_{t}+\varepsilon_{\mathrm{it}}$$ (1)

*Prosafety_it_* is the dependent variable (safety performance), where subscripts *i* and *t* denote firm and year, respectively. *α_0_* is the constant term, *β_1_ and β_2_* are the parameters to be estimated. *Report_it_* is the core independent variable, *Control_it_* represents a set of control variables, *Firm_i_* denotes the individual effect, *Year_t_* stands for the time effect, *ε_it_* signifies the random error term.

To examine the mediating effect of special reserve balance (*Srb*), the following test model consists of model 2 and model 3.

$$\mathrm{Sr}b_{\mathrm{it}}=\gamma_{0}+\gamma_{1}{\text{Report}}_{\mathrm{it}}+\gamma_{2}{\text{control}}_{\mathrm{it}}+{\text{Firm}}_{i}+{\text{Year}}_{t}+\varepsilon_{\mathrm{it}}$$ (2)

$$\begin{array}{c} \begin{array}{l} {\text{Prosafety}}_{\mathrm{it}}=\alpha_{0}+\alpha_{1}{\text{Report}}_{\mathrm{it}}+\alpha_{2}{\text{control}}_{\mathrm{it}}\\ +\alpha_{3}\mathrm{Sr}b_{\mathrm{it}}+{\text{Firm}}_{i}+{\text{Year}}_{t}+\varepsilon_{\mathrm{it}} \end{array} \end{array}$$ (3)

To examine the mediating effect of internal control quality, the following test model consists of model 4 and model 5.

$$\begin{array}{c} {\text{Incont}}_{\mathrm{it}}=\gamma_{0}+\gamma_{1}{\text{Report}}_{\mathrm{it}}+\gamma_{2}{\text{control}}_{\mathrm{it}}+{\text{Firm}}_{i}+{\text{Year}}_{t}+\varepsilon_{\mathrm{it}} \end{array}$$ (4)

$$\begin{array}{c} \begin{array}{l} {\text{Prosafety}}_{\mathrm{it}}=\alpha_{0}+\alpha_{1}{\text{Report}}_{\mathrm{it}}+\alpha_{2}{\text{control}}_{\mathrm{it}}\\ +\alpha_{3}{\text{Incont}}_{\mathrm{it}}+{\text{Firm}}_{i}+{\text{Year}}_{t}+\varepsilon_{\mathrm{it}} \end{array} \end{array}$$ (5)

Both *γ* and *α* are parameters to be estimated, while the remaining variables are defined as in model 1.

## 4 Results

### 4.1 Descriptive statistics

Table 2 presents the descriptive statistics for variables. The mean value of corporate whistleblower system *(Report)* is 1.649, ranging from 0 to 6.127, with a standard deviation of 1.431, indicating substantial variation across firms and years. The mean value of workplace fatalities *(Death_N)* is 0.276, corresponding to an average of 0.318 fatalities per firm-year before logarithmic transformation. The mean value of workplace fatality rate *(Death_R)* is 0.313 per 1,000 employees, with a maximum of 4.131 per 1,000 employees. The incidence of employee fatalities *(Death_D)* shows that 20.2% of sample firms experience at least one workplace fatality annually. The relatively large standard deviations and wide ranges across these safety indicators reveal considerable heterogeneity in workplace safety performance among enterprises, underscoring the persistent severity of safety challenges in high-risk industries. All control variables fall within reasonable ranges, and their standard deviations confirm sufficient variation across firms and years, indicating that our sample possesses high discriminatory power for empirical analysis.

**Table 2:** Descriptive statistics of key variables.

Variable	Obs	Mean	Std. Dev.	Min	Median	Max
*Report*	3,086	1.649	1.431	0.000	1.609	6.127
*Death_N*	3,086	0.276	0.566	0.000	0.000	2.398
*Death_R*	3,086	0.313	0.649	0.000	0.000	4.131
*Death_D*	3,086	0.202	0.375	0.000	0.000	1.000
*Srb*	3,086	1.174	0.796	0.000	1.563	2.387
*Incont*	3,086	1.975	0.306	0.000	2.027	2.328
*Size*	3,086	23.051	1.584	19.509	22.824	28.718
*Roa*	3,086	0.008	0.012	−0.019	0.006	0.054
*Tobin Q*	3,086	1.616	0.910	0.000	1.327	5.473
*Lev*	3,086	0.483	0.207	0.068	0.492	0.896
*Empl*	3,086	8.292	1.393	5.602	8.151	12.428
*Acci*	3,086	7.022	0.727	5.366	7.015	8.639
*Growth*	3,086	0.217	1.211	−2.683	0.082	33.726
*Boardsize*	3,086	2.268	0.185	0.000	2.303	2.944
*Indp*	3,086	37.626	6.183	0.000	33.330	80.000
*HHI*	3,086	0.104	0.061	−0.117	0.083	0.561

### 4.2 Benchmark regression

Table 3 presents the baseline regression results for the relationship between corporate whistleblower system and safety performance. Across columns (1) to (3), the coefficients on corporate whistleblower system (*Report*) are all consistently negative and statistically significant. In terms of economic magnitude, a one-standard-deviation increase in *Report* is associated with a 33.2% reduction in the number of workplace fatalities (*Death_N*), a 32.5% decrease in employee fatality rate (*Death_R*), and a 4.9-percentage-point change in the likelihood of workplace fatalities (*Death_D*). These results suggest that robust whistleblower system effectively mitigates safety risks, leading to lower employee fatality rate and a lower probability of workplace fatalities. Overall, the evidence confirms a significant positive association between corporate whistleblower system and safety performance, thereby supporting Hypothesis 1.

**Table 3:** Benchmarking regression analysis results.

Variable	(1)	(2)	(3)
	*Death_N*	*Death_R*	*Death_D*
*Report*	−0.064***	−0.071***	−0.034**
	(0.020)	(0.020)	(0.014)
*Lev*	−0.083	−0.192	−0.060
	(0.094)	(0.145)	(0.064)
*Tobin Q*	0.008	0.016	0.013
	(0.014)	(0.024)	(0.009)
*Roa*	0.644	0.720	0.870
	(0.968)	(1.395)	(0.662)
*Size*	0.026***	0.027***	0.011*
	(0.010)	(0.008)	(0.006)
*Empl*	−0.008	−0.235***	0.004
	(0.027)	(0.052)	(0.017)
*Acci*	0.021	−0.020	0.023
	(0.029)	(0.039)	(0.020)
*Boardsize*	0.007	−0.020	0.004
	(0.012)	(0.013)	(0.008)
*Indp*	0.003	0.003	0.002
	(0.004)	(0.004)	(0.002)
*Growth*	−0.023*	−0.015*	−0.020*
	(0.012)	(0.009)	(0.011)
*HHI*	−0.063	0.507	−0.116
	(0.295)	(0.465)	(0.202)
FE	Yes	Yes	Yes
Cons	0.117	2.434***	0.021
	(0.378)	(0.610)	(0.239)
*N*	3,086	3,086	3,086
*R* ^2^	0.269	0.217	0.273

***p < 0.01, **p < 0.05, *p < 0.1. Heteroscedasticity-robust standard error is provided in parentheses. Firm and Year FE included. Cons denotes the constant term. The same applies to subsequent tables.

### 4.3 Endogeneity tests

To mitigate potential endogeneity concerns, we employed a firm-level multi-period difference-in-differences (DID) test approach (46). Specifically, we defined a treatment dummy (*Treat*) that equals 1 for companies with whistleblower system (treatment group) and 0 otherwise (control group), alongside a time dummy (*Post*) that takes the value of 1 for the year of system implementation and all subsequent years, and 0 otherwise. The interaction term (*DID = Treat × Post*) serves as the primary explanatory variable in regressions. As shown in Columns (1)–(3) of Table 4, the coefficients on *DID* remain statistically significant, albeit with some variations in magnitude relative to baseline estimates.

**Table 4:** Endogeneity tests: DID and lagged dependent variable.

Variable	DID			Lagged dependent variable		
	(1)	(2)	(3)	(4)	(5)	(6)
	*Death_N*	*Death_R*	*Death_D*	*Death_N*	*Death_R*	*Death_D*
*DID*	−0.082**	−0.108***	−0.057**			
	(0.039)	(0.032)	(0.026)			
*Report*				−0.034*	−0.040*	−0.032**
				(0.019)	(0.021)	(0.013)
Controls	Yes	Yes	Yes	Yes	Yes	Yes
FE	Yes	Yes	Yes	Yes	Yes	Yes
Cons	0.029	2.132***	−0.025	0.285	0.914	0.264***
	(0.378)	(0.576)	(0.239)	(0.266)	(0.621)	(0.023)
*N*	3,086	3,086	3,086	2,580	2,580	2,580
*R* ^2^	0.267	0.211	0.272	0.284	0.215	0.276

^***^p < 0.01, ^**^p < 0.05, ^*^p < 0.1. Heteroscedasticity-robust standard error is provided in parentheses. Controls represents control variables. The same applies to the tables below.

Building on this, we further verified the parallel trend assumption via an event study, setting the year before implementation as the baseline. In our sample, a total of 1,860 firm-year observations belonged to firms that never adopted whistleblower system during the entire 2013–2024 sample period. These observations served as our never-treated control group. Firms that adopted the system in later years were treated as controls prior to their adoption and then switched to treatment status thereafter, contributing to the estimation as not-yet-treated controls. Figure 3 presents the estimation results. The coefficients for all pre-implementation periods are statistically insignificant, confirming that the treatment and control groups followed parallel trends prior to the intervention, thereby satisfying the parallel trend assumption required for our DID estimation.

**Figure 3:**
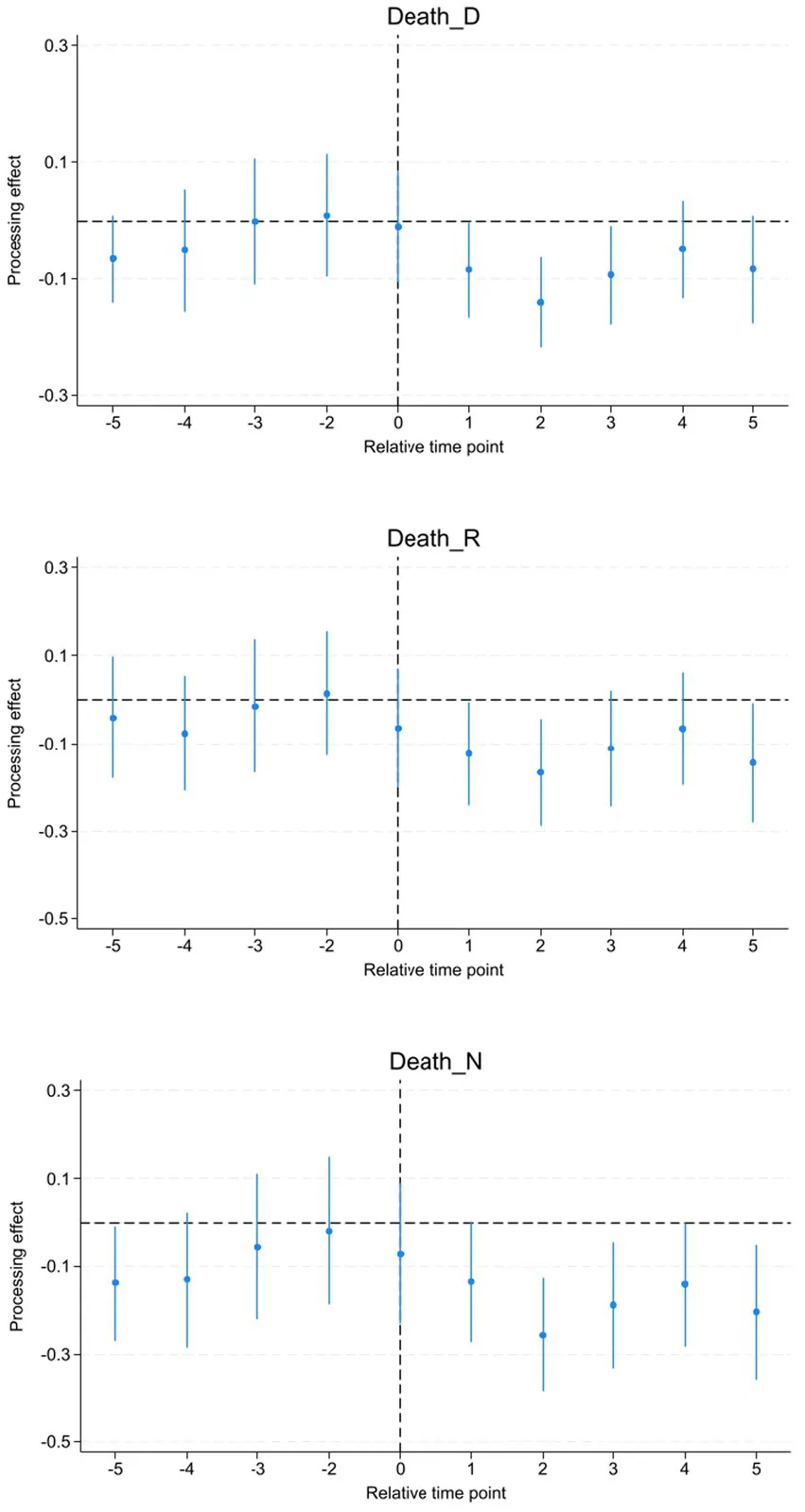
Results of parallel trends test.

Furthermore, we lag the dependent variable by two periods, and the results presented in columns (4)–(6) align with our baseline findings. Collectively, these tests confirm that our results are robust to potential endogeneity.

### 4.4 Robustness tests

#### 4.4.1 Double machine learning

To mitigate the curse of dimensionality and model misspecification arising from the high-dimensional determinants of safety performance, we adopted the Double Machine Learning (DML) approach for causal inference. In accordance with Chernozhukov et al. (47), we construct the following model 6:

$$\begin{array}{c} \begin{array}{l} {\text{Prosafety}}_{\mathrm{it}}=\theta_{0}{\text{Report}}_{\mathrm{it}}+g(X_{\mathrm{it}})\\ +U_{\mathrm{it}},E(U_{\mathrm{it}}|{\text{Report}}_{\mathrm{it}},X_{\mathrm{it}})=0 \end{array} \end{array}$$ (6)

Where *θ_0_* is the causal inference coefficient. *X_it_* denotes the set of control variables, *g(X_it_)* specifies the functional form through which these control variables influence safety performance, and *U_it_* is the error term. Other variables are defined as in model1.

To address the regularization bias arising from directly applying DML estimation, we further employ model 7 as an auxiliary regression:

$$\begin{array}{c} {\text{Report}}_{\mathrm{it}}=m(X_{\mathrm{it}})+V_{\mathrm{it}},E(V_{\mathrm{it}}|X_{\mathrm{it}})=0 \end{array}$$ (7)

Where *m(X_it_)* represents the functional form of control variables’ impact on corporate whistleblower system, and *V_it_* is the error term. Both the main and auxiliary regressions are estimated using Random Forests.

We implement 5-fold cross-fitting with 10 repeated sample splits to reduce sensitivity to random partitioning and enhance estimate stability. Random Forest Algorithm (RFA) is employed as the base learner for both the treatment and outcome stages. Hyperparameters (e.g., the number of trees, maximum depth, and minimum node size) are tuned via grid search combined with 5-fold cross-validation.

Columns (1)–(4) of Table 5 demonstrate that, after employing DML method to control for high-dimensional confounders and potential nonlinearity, the negative relationship between corporate whistleblower system (*Report*) and the number of workplace fatalities (*Death_N*) and employee fatality rate (*Death_R*) remains statistically significant. This confirms that the relationship between corporate whistleblower system and improved safety performance is robust.

**Table 5:** Endogeneity tests: double machine learning.

Variables	(1)	(2)	(3)	(4)	(5)	(6)
	*Death_N*	*Death_N*	*Death_R*	*Death_R*	*Death_D*	*Death_D*
*Report*	−0.052*	−0.060*	−0.048*	−0.066**	−0.021	−0.032*
	(0.028)	(0.031)	(0.025)	(0.028)	(0.018)	(0.019)
Control variable term	Yes	Yes	Yes	Yes	Yes	Yes
The quadratic term of the control variable	No	Yes	No	Yes	No	Yes
FE	Yes	Yes	Yes	Yes	Yes	Yes

^**^p < 0.05, ^*^p < 0.1. Heteroscedasticity-robust standard error is provided in parentheses.

#### 4.4.2 Changing model and winsorization

Given that *Death_N* is a count variable with a high proportion of zeros, we adopted the Poisson Pseudo-Maximum Likelihood (PPML) model. For the non-negative rate variable *Death_R*, we employ the PPML model with an offset specification based on the number of employees. Finally, to account for the binary nature of *Death_D*, we use a fixed-effects logit model (48). Additionally, to mitigate potential estimation bias driven by extreme outliers, we winsorized the dependent variable at the 1st and 99th percentiles to limit the influence of extreme outliers without sacrificing statistical power (49). As presented in Table 6, the negative coefficient of corporate whistleblower system (*Report*) on workplace fatalities (*Death_N*) and employee fatality rate (*Death_R*) remains statistically significant, reinforcing our baseline findings.

**Table 6:** Robustness tests based on changing model and winsorization.

Variable	Changing model			Winsorization		
	(1)	(2)	(3)	(4)	(5)	(6)
	*Death_N*	*Death_R*	*Death_D*	*Death_N*	*Death_R*	*Death_D*
*Report*	−0.187***	−0.385***	−0.201*	−0.061***	−0.069***	−0.029**
	(0.069)	(0.116)	(0.111)	(0.020)	(0.019)	(0.014)
Controls	Yes	Yes	Yes	Yes	Yes	Yes
FE	Yes	Yes	Yes	Yes	Yes	Yes
Cons	−1.035	5.405**		0.298	2.463***	0.055
	(1.269)	(2.393)		(0.320)	(0.563)	(0.204)
*N*	3,086	3,086	3,086	3,086	3,086	3,086
*R* ^2^	0.208	0.164	0.029	0.269	0.215	0.273

^***^p < 0.01, ^**^p < 0.05, ^*^p < 0.1. Heteroscedasticity-robust standard error is provided in parentheses.

#### 4.4.3 Replacing variables and changing fixed effects

To ensure the robustness of our baseline results, we adopted alternative variable measures and a high-dimensional fixed-effects specification. First, we construct an alternative proxy for safety performance, the Fatalities per Billion Revenue Ratio (*FBRR* = the number of employee fatalities/annual revenue in billions of CNY). Second, we redefine whistleblower system using a dummy variable (*Report_W*), which equals 1 if keywords (e.g., “reporting,” “whistleblowing,” and “whistleblower”) are identified in corporate reports and 0 otherwise. Furthermore, following Ayaz et al. (46), we replaced the original firm-year fixed effects with province-year fixed effects to better absorb region-specific time-varying shocks. The results presented in Table 7 indicate that the relationship between corporate whistleblower system and safety performance remains robust.

**Table 7:** Robustness tests: replacing variables and high-dimensional fixed effects.

Variable	Replacing variables				Changing fixed effects		
	(1)	(2)	(3)	(4)	(5)	(6)	(7)
	*Death_N*	*Death_R*	*Death_D*	*FBRR*	*Death_N*	*Death_R*	*Death_D*
*Report_W*	−0.094**	−0.137***	−0.059**				
	(0.046)	(0.044)	(0.030)				
*Report*				−0.005**	−0.027***	−0.042***	−0.015**
				(0.002)	(0.010)	(0.012)	(0.007)
Controls	Yes	Yes	Yes	Yes	Yes	Yes	Yes
Firm/Time Fixed Effects	Yes	Yes	Yes	Yes	No	No	No
Industry fixed effects	No	No	No	No	Yes	Yes	Yes
Cons	0.042	2.355***	−0.295	0.351	−1.005***	1.932*	−0.698***
	(0.377)	(0.606)	(0.226)	(0.244)	(0.251)	(0.566)	(0.164)
*N*	3,086	3,086	3,086	3,086	3,086	3,086	3,086
*R* ^2^	0.268	0.217	0.264	0.119	0.142	0.211	0.133

^***^p < 0.01, ^**^p < 0.05, ^*^p < 0.1. Heteroscedasticity-robust standard error is provided in parentheses.

### 4.5 Mechanism test

#### 4.5.1 Mediating effect of safety investment

We included special reserve balance (*Srb*) as a mediator in the regression analysis, and the regression results are reported in Table 8. Columns (1)–(4) show that corporate whistleblower system (*Report*) significantly correlates with safety performance and special reserve balance (*Srb*), and Columns (5)–(7) suggest that both *Report* and *Srb* significantly correlate with *Death_N, Death_R* and *Death_D*. The three-step mediation analysis results confirm the mediating role of special reserve balance in the relationship between corporate whistleblower system and safety performance, thereby supporting Hypothesis 2.

**Table 8:** Results of the mediation of safety investment.

Variable	(1)	(2)	(3)	(4)	(5)	(6)	(7)
	*Death_N*	*Death_R*	*Death_D*	*Srb*	*Death_N*	*Death_R*	*Death_D*
*Report*	−0.064***	−0.071***	−0.034**	−0.022*	−0.063***	−0.070***	−0.033**
	(0.020)	(0.020)	(0.014)	(0.012)	(0.020)	(0.020)	(0.014)
*Srb*					0.047**	0.005*	0.030*
					(0.023)	(0.003)	(0.017)
Controls	Yes	Yes	Yes	Yes	Yes	Yes	Yes
FE	Yes	Yes	Yes	Yes	Yes	Yes	Yes
Cons	0.117	2.434***	0.021	0.584**	0.439	0.066	0.274
	(0.378)	(0.610)	(0.239)	(0.259)	(0.453)	(0.048)	(0.296)
*N*	3,086	3,086	3,086	3,086	3,086	3,086	3,086
*R* ^2^	0.269	0.217	0.273	0.825	0.270	0.219	0.274

^***^p < 0.01, ^**^p < 0.05, ^*^p < 0.1. Heteroscedasticity-robust standard error is provided in parentheses. Special Reserve balance (Srb) is a reverse proxy for actual safety investment, where a larger Srb indicates lower safety investment.

To formally test the mediating role of special reserve balance, we employed a bootstrap procedure with 300 replications to construct bias-corrected 95% confidence intervals (BC CIs) for the indirect effects. The estimated indirect effects of special reserve balance are −0.0011 (*SE* = 0.0009, 95% *CI* = [−0.0035, −0.0001]) for *Death_N*, −0.0001 (*SE* = 0.0001, 95% *CI* = [−0.0003, −0.0001]) for *Death_R*, and −0.0012 (*SE* = 0.0011, 95% *CI* = [−0.0050, −0.0002]) for *Death_D*. Since none of these confidence intervals contain zero, the indirect effects of special reserve balance are statistically significant, confirming that special reserve balance serves as a mediator through which corporate whistleblower system positively correlates with safety performance. These results further support Hypothesis 2.

#### 4.5.2 Mediating effect of internal control quality

Given that internal control quality (*Incont*) includes observations with values more than three standard deviations below the mean, we adopted a trimming procedure to exclude these extreme observations, thus minimizing the influence of outliers on our estimation. This approach preserves the integrity of the original data distribution while mitigating the influence of outliers, which retained 3,023 firm-year observations. Given the small proportion of trimmed observations (2.0% of the full sample), this trimming procedure is unlikely to materially affect the representativeness of our full observations. Then, we included internal control quality (*Incont*) as the mediator in mediation regressions, with results presented in Table 9. Columns (1)–(4) show that *Report* significantly correlates with safety performance and *Incont,* and Columns (5)–(7) suggest that both *Report* and *Incont* significantly correlate with *Death_N, Death_R* and *Death_D*. These results confirm the mediating role of internal control quality in the relationship between corporate whistleblower systems and safety performance, thereby supporting Hypothesis 3.

**Table 9:** Results of the mediation of internal control quality.

Variables	(1)	(2)	(3)	(4)	(5)	(6)	(7)
	*Death_N*	*Death_R*	*Death_D*	*Incont*	*Death_N*	*Death_R*	*Death_D*
*Report*	−0.064^***^	−0.071^***^	−0.034**	0.006*	−0.060***	−0.007***	−0.030**
	(0.020)	(0.020)	(0.014)	(0.003)	(0.021)	(0.002)	(0.014)
Incont					−0.280***	−0.034***	−0.172***
					(0.058)	(0.008)	(0.031)
Controls	Yes	Yes	Yes	Yes	Yes	Yes	Yes
FE	Yes	Yes	Yes	Yes	Yes	Yes	Yes
Cons	0.117	2.434^***^	0.021	1.783***	0.670*	0.127**	0.326
	(0.378)	(0.610)	(0.239)	(0.072)	(0.399)	(0.050)	(0.251)
*N*	3,086	3,086	3,086	3,023	3,023	3,023	3,023
*R* ^2^	0.269	0.217	0.273	0.056	0.283	0.248	0.285

^***^p < 0.01, ^**^p < 0.05, ^*^p < 0.1. Heteroscedasticity-robust standard error is provided in parentheses.

We employed the same bootstrap procedure with 300 replications to formally test the indirect effects of internal control quality. The estimated indirect effects of internal control quality are −0.0018 (*SE* = 0.0011, 95% *CI* = [−0.0044, −0.0001]) for *Death_N*, −0.0002 (*SE* = 0.0001, 95% *CI* = [−0.0006, −0.0001]) for *Death_R*, and −0.0011 (*SE* = 0.0006, 95% *CI* = [−0.0027, −0.0001]) for *Death_D*. All three bootstrap confidence intervals exclude zero, indicating that the indirect effects are statistically significant. These results confirm that whistleblower system positively correlates with safety performance through the mediating role of internal control quality, thereby further supporting Hypothesis 3.

### 4.6 Heterogeneity analysis

#### 4.6.1 Regional heterogeneity

The effectiveness of corporate whistleblower system may vary substantially across regions. We split the sample into Eastern region and Central & Western region firms based on registration location to examine the heterogeneous relationship between corporate whistleblower system and safety performance. The group regression results are reported in Table 10. We find that whistleblower system has a significantly positive relationship with safety performance for firms located in the Eastern region. In contrast, the estimated effects are mostly insignificant for firms in Central & Western regions, with only the coefficient of *Report* on *Death_R* remaining significant. Furthermore, we used the bootstrap inter-group coefficient difference (Bdiff) test method to test whether the difference between the group coefficients of group regression is significant. Three empirical *p*-values for the difference between two groups are significant at the 10% (*Death_N* and *Death_D*) or 5% level (*Death_R*) (see Table 10), indicating that corporate whistleblower system has a stronger relationship with safety performance in the Eastern region.

**Table 10:** Heterogeneity analysis by corporate regional location.

Variable	ER	CWR	ER	CWR	ER	CWR
	(1)	(2)	(3)	(4)	(5)	(6)
	*Death_N*	*Death_N*	*Death_R*	*Death_R*	*Death_D*	*Death_D*
*Report*	−0.100***	−0.038	−0.116***	−0.041*	−0.052***	−0.022
	(0.030)	(0.027)	(0.037)	(0.021)	(0.019)	(0.018)
Controls	Yes	Yes	Yes	Yes	Yes	Yes
FE	Yes	Yes	Yes	Yes	Yes	Yes
Cons	0.612	0.233	1.889***	3.056***	0.297	−0.041
	(0.481)	(0.449)	(0.658)	(0.884)	(0.315)	(0.284)
*N*	1715	1,371	1715	1,371	1715	1,371
*R* ^2^	0.239	0.298	0.159	0.250	0.257	0.294
Empirical *p*-value (Bdiff)	0.072		0.030		0.096	

^***^p < 0.01, ^**^p < 0.05, ^*^p < 0.1. Heteroscedasticity-robust standard error is provided in parentheses. ER, Eastern Regions; CWR, Central and Western Regions.

A plausible explanation is that the Eastern region generally features higher economic development and more stringent workplace safety supervision. As a result, local firms respond more actively to national policies, thereby strengthening the implementation and governance effectiveness of whistleblower systems. This evidence is also consistent with the conclusion documented by Wang and Mao (50), who reports that workplace safety governance effectiveness weakens gradually from the Eastern region to the Central & Western region.

#### 4.6.2 Ownership heterogeneity

State-owned enterprises (SOEs) and Private enterprises (PEs) differ markedly in governance structures, resource endowments and business objectives (51). The effectiveness of whistleblower system may thus vary across ownership types. We partition the sample into SOEs and PEs subsamples based on ownership type and perform subsample regressions. Table 11 presents the heterogeneity test results based on the nature of enterprise property rights. Nevertheless, the *p*-values for the difference between two groups are 0.168, 0.330, and 0.490 (larger than 0.1). These Bdiff test findings indicate that corporate whistleblower system negatively correlates with *Death_N*, *Death_D*, and *Death_R* in both SOEs and PEs, suggesting that the effectiveness of whistleblower systems in reducing workplace fatalities is robust across ownership structures, though the magnitude of the effect may differ.

**Table 11:** Heterogeneity analysis by ownership structure.

Variable	SOEs	PEs	SOEs	PEs	SOEs	PEs
	(1)	(2)	(3)	(4)	(5)	(6)
	*Death_N*	*Death_N*	*Death_R*	*Death_R*	*Death_D*	*Death_D*
*Report*	−0.063**	−0.047*	−0.049**	−0.079*	−0.029*	−0.032*
	(0.026)	(0.026)	(0.021)	(0.038)	(0.017)	(0.019)
Controls	Yes	Yes	Yes	Yes	Yes	Yes
FE	Yes	Yes	Yes	Yes	Yes	Yes
Cons	0.445	0.150	3.192***	1.790***	0.133	0.010
	(0.515)	(0.339)	(0.874)	(0.693)	(0.315)	(0.240)
*N*	1845	1,241	1845	1,241	1845	1,241
*R* ^2^	0.286	0.172	0.292	0.162	0.288	0.179
Empirical *p*-value (Bdiff)	0.168		0.330		0.490	

^***^p < 0.01, ^**^p < 0.05, ^*^p < 0.1. Heteroscedasticity-robust standard error is provided in parentheses. SOEs, state-owned enterprises; PEs, private enterprises.

## 5 Discussion and conclusion

This study investigates whether and how corporate whistleblower system affects workplace safety performance using a sample of Chinese A-share listed companies in high-risk industries from 2013 to 2024. Three main conclusions emerge. First, whistleblower systems negatively correlate with workplace fatalities and employee mortality rates. This result withstands a range of robustness checks. Second, the safety governance effect operates through two pathways: safety investment and internal control quality. Whistleblower systems deter non-compliance by raising the expected costs of violations, prompting firms to increase realized safety expenditures (as reflected in lower special reserve balances). Simultaneously, they correlate with internal control quality by facilitating the upward transmission of hazard information from frontline employees to management, enabling more targeted risk responses. Third, the relationship is heterogeneous across regions: it is more pronounced in the Eastern regions than in the Central & Western regions. However, it does not differ significantly between state-owned and private enterprises, suggesting that the effectiveness of whistleblower systems in reducing workplace fatalities is robust across ownership structures.

### 5.1 Theoretical implications

This study makes three theoretical contributions. First, we extend the governance boundary of whistleblowing by revealing the conditional nature of its governance logic. Prior whistleblowing research has predominantly operated within financial and compliance contexts, concentrated on fraud (7), financial misreporting (9, 17), and ethical violations (11). Workplace safety, a domain where hazards are characterized by specialization, concealment, and dynamism and are therefore most dependent on internal information discovery (5), has received little attention. By demonstrating that whistleblower system is associated with improved safety outcomes, we bridge these two streams of literature and establish whistleblowing as a cross-cutting governance mechanism that extends well beyond financial compliance (8, 10). In doing so, we show that the safety context does not simply replicate the financial governance logic but fundamentally transforms it. Unlike financial misconduct, safety risk identification relies heavily on frontline employees’ on-site judgment and technical expertise (5, 6); the consequences of safety violations exhibit temporal lags, creating a misalignment between short-term gains and long-term risks (31); and the concealment of hazards makes them resistant to external regulatory oversight (5, 16). These features make safety governance more dependent on employee proactivity, timely management responses, and substantive resource commitments—than in the financial domain (8, 10). By revealing these contextual distinctions, this study advances whistleblowing theory from a one-size-fits-all logic toward a context-sensitive framework that acknowledges the distinct operational demands of different domains of organizational misconduct.

Second, we illuminate the theoretical pathway from behavioral deterrence to accident prevention. Prior studies have acknowledged the governance benefits of whistleblowing but left the underlying mechanisms under-examined (9). Guided by deterrence theory (22, 23) and signaling theory (24, 25), this study identifies and validates two complementary pathways: a resource pathway (safety investment) and a governance pathway (internal control quality). The former reveals how whistleblower systems raise the expected costs of non-compliance, prompting firms to increase preventive safety investments (19, 33); the latter reveals how whistleblower systems signal organizational commitment to safety, encouraging voluntary hazard whistleblowing and strengthening the information and communication function of internal controls (34, 35). Together, these pathways explicate the complete theoretical mechanism from deterrence to accident prevention, filling the theoretical gap regarding “how whistleblower systems generate governance effects” and responding to calls for disaggregated mechanism analysis in whistleblowing research (8).

Third, this study builds on a multi-level theoretical framework. It integrates accident causation theory (20, 21), deterrence theory (22, 23), and signaling theory (24, 25) into a unified analytical framework. Accident causation theory explains why accidents occur, deterrence theory explains why firms respond, and signaling theory explains why employees report, which correspond to different stages of the pathway and form a complete explanatory logic. This integrated framework transcends existing studies’ reliance on single theories and offers a more powerful theoretical tool for understanding the governance effects of whistleblower systems. Concurrently, this study shifts the analytical focus from external regulators to internal employees, thus revealing employees’ unique value in safety governance.

### 5.2 Practical implications

Based on these findings, we propose the following targeted recommendations. First, enterprises should move beyond formal compliance and build actionable, incentive-aligned whistleblower system. This includes establishing anonymous and accessible whistleblowing channels, offering clear rewards and protection against retaliation, and embedding whistleblowing into corporate safety culture through regular training and advocacy. Second, a synergistic framework combining internal whistleblowing with external regulation should be developed to ensure that reported hazards translate into realized safety expenditures rather than merely increasing reserve appropriations. Firms should integrate whistleblower mechanisms with existing risk control and hazard screening systems to create a closed-loop process of whistleblowing, verification, rectification, and expenditure. Regulators should use whistleblower clues as key audit evidence to prevent misappropriation of safety funds. Third, firms should leverage whistleblower system to enhance internal control quality, thereby shifting safety management from surface compliance to genuine risk reduction. By incorporating whistleblower information into internal control risk assessments and establishing feedback loops that track rectification outcomes, enterprises can ensure that every reported hazard receives timely and effective action. Lastly, a differentiated policy approach is warranted. Given that the safety governance effects of whistleblower systems are more pronounced in Eastern region, a differentiated regional policy approach is warranted. By contrast, the effect does not vary significantly between SOEs and PEs, suggesting that mandatory whistleblower system adoption could be expected to benefit both ownership types. At the same time, all firms require tailored guidance to embed whistleblower mechanisms across their entire production chain, thereby creating a dual-layer safety safeguard that combines external regulatory pressure with internal self-governance.

### 5.3 Limitations and future directions

Despite the theoretical and practical implications of this study, several limitations should be acknowledged. First, our corporate whistleblower system measure was based on keyword frequency in corporate reports, which captures disclosure rather than the actual existence, usage, or effectiveness of whistleblower systems. Although we conducted manual validation, this measure does not distinguish between substantive and symbolic adoption. It also cannot capture channel trustworthiness, anti-retaliation protection, or management responsiveness. Future research could employ survey-based measures to capture the quality and functionality of whistleblower systems. Second, although we employed multiple identification strategies, including firm fixed effects, staggered DID, and DML, our findings remain correlational rather than causal. Unobserved confounders, such as safety culture or managerial risk preferences, may still bias estimates. Future studies could leverage quasi-experimental designs, for instance regulatory shocks mandating whistleblower system adoption, to strengthen causal inference. Third, employee-level mechanisms remain untested. Our theoretical framework incorporates signaling theory, which involves employee perceptions, trust in whistleblowing channels, and actual whistleblowing behavior. However, our archival data do not permit direct measurement of these micro-level processes. We therefore focus on organizational responses, safety investment and internal control, as necessary conditions for governance effects. Future research could use surveys, experiments, or case studies to directly test the psychological and behavioral mechanisms underlying whistleblower effectiveness. Fourth, our fatality data are drawn from publicly available sources, which may suffer from underreporting, particularly for minor accidents. While fatalities are generally less susceptible to underreporting than non-fatal injuries due to their materiality and regulatory disclosure requirements, the possibility of incomplete reporting remains. In addition, the inclusion of contractor fatalities in the civil engineering construction sector (E01) may introduce measurement imprecision in the fatality rate (*Death_R*), as the number of employees may not perfectly reflect the exposure base for contractor workers. While this concern is largely confined to the E01 subsector, the lack of granular employment data—such as separate counts for direct employees versus contractors—may introduce measurement noise. Future research could address these limitations by supplementing public data with regulatory inspection records or insurance claims, and by employing more detailed labor force classification data to refine the measurement of fatality rates. Lastly, the sample is limited to A-share listed companies in Chinese high-risk industries. These firms are subject to mandatory safety disclosure requirements and regulatory oversight that may not apply to unlisted firms or firms in other institutional environments. The governance effects of whistleblower systems may differ in contexts with weaker regulatory enforcement, different cultural norms around whistleblowing, or alternative corporate governance structures. Cross-country comparative studies or examinations of unlisted firms would enhance generalizability.

## Data Availability

Publicly available datasets were analyzed in this study. This data can be found here: 10.17632/tbsrh4vmgw.1.
