# Supplementary material for: Corporate whistleblower system and workplace safety performance: from the dual perspectives of safety investment and internal control
Source: Front Public Health. 2026 Sep 23;14:1935312. doi: 10.3389/fpubh.2026.1935312 (PMC13642825; doi:10.3389/fpubh.2026.1935312)
Supplement: Supplementary file 1 [file Supplementary_file_1.DOCX]

# Appendix A. Construction and Validation of the Keyword Dictionary for Whistleblower Systems

**A.1 Dictionary Construction**

Using the similar-word vector library on the WINGO financial platform, we constructed a keyword dictionary for corporate whistleblower systems through the following four-step procedure:

**(1) Seed word extraction based on definitions.** Drawing on the *Basic Standards for Enterprise Internal Control* (2008), the *Opinions of the General Office of the State Council on Further Strengthening the Protection of the Lawful Rights and Interests of Small and Medium Investors in the Capital Market* (2013), the *Guiding Opinions on Strengthening and Regulating In-process and Post-event Supervision* (2019), and theoretical definitions of internal reporting mechanisms in prior literature (1, 2), we extracted five core seed words for corporate whistleblower systems: “举报”、”吹哨”、”揭发”、”举报人”、”吹哨人”.

**(2) Word vector expansion.** Using the deep learning similarity-word module on the WINGO financial platform, we obtained semantically similar words for each seed word. For each seed word, we selected the top 30 words with the highest semantic similarity as candidate similar words, recording each similar word and its similarity score.

**(3) Seed word refinement and iterative expansion.** Recognizing that the five seed words alone may not fully capture all expressions of whistleblower systems in corporate disclosure texts, we added similar words with similarity scores greater than 0.7 as new seed words (3) and repeated step (2) until no new valid similar words could be generated.

**(4) Manual screening and denoising.** Given that deep learning algorithms operate at the machine-language level and cannot precisely identify words relevant to corporate whistleblower systems, we manually screened out similar words unrelated to whistleblower systems. Two independent researchers reviewed the candidate word pool and, based on the conceptual definition of whistleblower systems, identified and marked words that were semantically irrelevant. For words marked as potentially irrelevant, we further traced back to the original texts—including annual reports, corporate social responsibility (CSR) reports, and internal control self-assessment reports—to retrieve the actual usage context of each word. For ambiguous words (e.g., “投诉”, “反映”, “申诉”, “建言”, “上报”), we established co-occurrence rules: these words were counted as whistleblower-system-related only when they co-occurred in context with words such as “违法”, “违规”, “违纪”, “隐患”, “不安全”, or “危险”.

**A.2 Validation of the Keyword-Based Measure**

To validate our measure, we manually coded a random subsample of 200 firm-year observations. Two research assistants independently reviewed the original reports and judged whether each firm had disclosed a whistleblower system. Inter-coder agreement exceeded 92%. Against this manually coded benchmark, our keyword-based measure achieved a precision rate of 89%, confirming that the dictionary reasonably captures the presence of whistleblower system disclosures.

1. Rani U, Pramudyastuti O, Nugraheni A. How Do Indonesian Listed Companies Disclose Information Related to Whistleblowing? *Jurnal Dinamika Akuntansi dan Bisnis* (2022); 9: 223-240.

2. Dyck A, Morse A, Zingales L. Who Blows the Whistle on Corporate Fraud? *The Journal of Finance* (2010); 65: 2213-2253.

3. Zhu M, Hu H. Identification of Performative Esg Behaviors Using Explainable Machine Learning. *Computational Economics* (2026); *DOI:10.1007/S10614-026-11319-6.*
